# The Vital Role of Immunohematology in Diagnosing Paroxysmal Cold Hemoglobinuria: A Clinical Case Report

**DOI:** 10.7759/cureus.69362

**Published:** 2024-09-13

**Authors:** Willy Nava Gutiérrez, Paola Villa Cortés, Ricardo Ramírez Morales, Juan P Espinoza Garza, Jaime E Espinosa Mora

**Affiliations:** 1 Hematology and Oncology, Instituto de Seguridad y Servicios Sociales de los Trabajadores del Estado de Nuevo León (ISSSTELEON), Monterrey, MEX; 2 Hematology, Universidad de Monterrey, Monterrey, MEX; 3 Critical Care Medicine, Instituto de Seguridad y Servicios Sociales de los Trabajadores del Estado de Nuevo León (ISSSTELEON), Monterrey, MEX; 4 Rheumatology, Instituto de Seguridad y Servicios Sociales de los Trabajadores del Estado de Nuevo León (ISSSTELEON), Monterrey, MEX; 5 Pulmonology, Instituto de Seguridad y Servicios Sociales de los Trabajadores del Estado de Nuevo León (ISSSTELEON), Monterrey, MEX; 6 Infectious Disease, Instituto de Seguridad y Servicios Sociales de los Trabajadores del Estado de Nuevo León (ISSSTELEON), Monterrey, MEX

**Keywords:** anca-negative, autoimmune hemolytic anemia (aiha), autoimmune vasculitis, cold agglutinin disease, complement-mediated hemolysis, eculizumab, mexico, paroxysmal cold hemoglobinuria (pch), rituximab, therapeutic plasma exchange (tpe)

## Abstract

Paroxysmal cold hemoglobinuria (PCH) is a rare autoimmune hemolytic anemia caused by the binding of IgG immunoglobulins to red blood cells at cold temperatures, leading to hemolysis upon rewarming. The Donath-Landsteiner test can show biphasic hemolysis, leading to diagnosis. There is no consensus, but chemoimmunotherapy with or without plasma exchange is commonly employed. We present the case of a 42-year-old male who experienced symptoms of hemolysis after cold exposure in a semi-arid, warm-climate city.

## Introduction

The origin of cold-induced hemolysis can be traced back to the mid-19th century [1-3]. In 1904, Julius Donath and the Nobel Prize-winning medical researcher Karl Landsteiner conducted a groundbreaking experiment. They observed that plasma, incubated at 5 and 37 degrees Celsius (°C) for 30 and 180 minutes, respectively, from patients with hemoglobinuria, induced in vitro hemolysis of both autologous and allogeneic red blood cells from healthy individuals. This test was named the Donath-Landsteiner test (DLT) in honor of its creators. This seminal experiment marked the identification of the first immune-mediated hemolytic anemia: paroxysmal cold hemoglobinuria (PCH) [1-3]. Nearly six decades later, Philip Levine provided evidence of the involvement of the P blood group antigen in the hemolytic reaction in PCH [4].

PCH constitutes 1% of autoimmune hemolytic anemias [5]. The annual incidence is estimated at 0.04 cases per 100,000 inhabitants, with over 50% of cases occurring in patients aged five years or younger. There is a secondary peak between the ages of 50 and 80, representing 21% of affected individuals [6].

The most frequent clinical presentation is hemoglobinuria, observed in 69% of cases, followed by jaundice (27%), fever (26%), weakness/fatigue (23%), pallor (17%), and abdominal pain (17%). In the majority of cases, there was a history of respiratory tract infection without a clear association with any specific causal agent. Concomitant neoplasms were more frequently observed in individuals aged 58 and above [6].

Despite the absence of therapeutic consensus, the common approach involves supportive care and the administration of chemoimmunotherapy with or without plasma exchange (PEX). Unlike warm autoimmune hemolytic anemia, PCH has a poor response to steroids [5,6].

We present the case of a middle-aged male who exhibited symptoms of direct antiglobulin test (DAT) negative hemolytic anemia with a positive Donath-Landsteiner test following cold exposure in Monterrey, a semi-arid, warm climate city in Mexico.

## Case presentation

This is the case of a 42-year-old male patient with a 15-year history of diabetes and hypertension. He had a previous hospitalization in January 2020 for antineutrophil cytoplasmic antibody (ANCA)-negative vasculitis, accompanied by rapidly progressive pauci-immune glomerulonephritis. The treatment during that hospitalization included steroids, hemodialysis, PEX, and red blood cell transfusions. This was followed by the ambulatory administration of rituximab (RTX) and mycophenolic acid which continued until May 2023.

**Figure 1:**
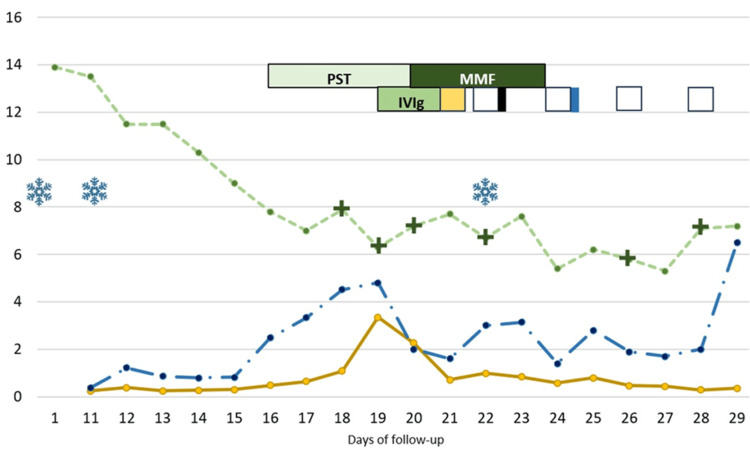
Patient's Follow-up and Treatment. X axis, days of treatment; Y axis, green line for hemoglobin in milligrams per deciliter (mg/dL), yellow line for lactic dehydrogenase in International Units per milliliter (UI/mL), blue line for total bilirubin in mg/dL; IVIg, intravenous immunoglobulin; MMF, mycophenolic acid; PST, pulse steroid; snowflakes, cold temperature exposure; green cross, red blood cell transfusion; yellow square, PEX with fresh frozen plasma; white square, albumin and saline solution PEX; black rectangle, cyclophosphamide (Cy); blue rectangle, RTX. The full description is provided in the text.

He presented at the rheumatology clinic on May 12th, 2023 (day 1) with fatigue and weakness, a non-productive cough leading to progressive dyspnea with moderate exertion, and a fever of 39-40°C without a specific time pattern. Consequently, he was referred to the emergency department. Initially, treatment was administered for community-acquired pneumonia, using ceftriaxone and clarithromycin. Chest angiotomography revealed peripheral infiltrates in the right apex and both lung bases with a tendency to consolidation. Subsequently, coverage for Pneumocystis jirovecii pneumonia was added. Blood and urine bacterial and fungal cultures, polymerase chain reaction for mycobacteria, Coccidioides spp, and pneumonia panel were conducted, and all yielded negative results. Neoplastic and sexually transmitted diseases were also ruled out. However, despite antibiotic therapy, there was no clinical improvement, prompting a bronchoscopy on May 24th, 2023 (day 11) in an operating room with an ambient temperature of 16°C (Figure 1), revealing erythematous mucosa and clear secretions in the right bronchial tree, with no other abnormalities.

**Figure 2:**
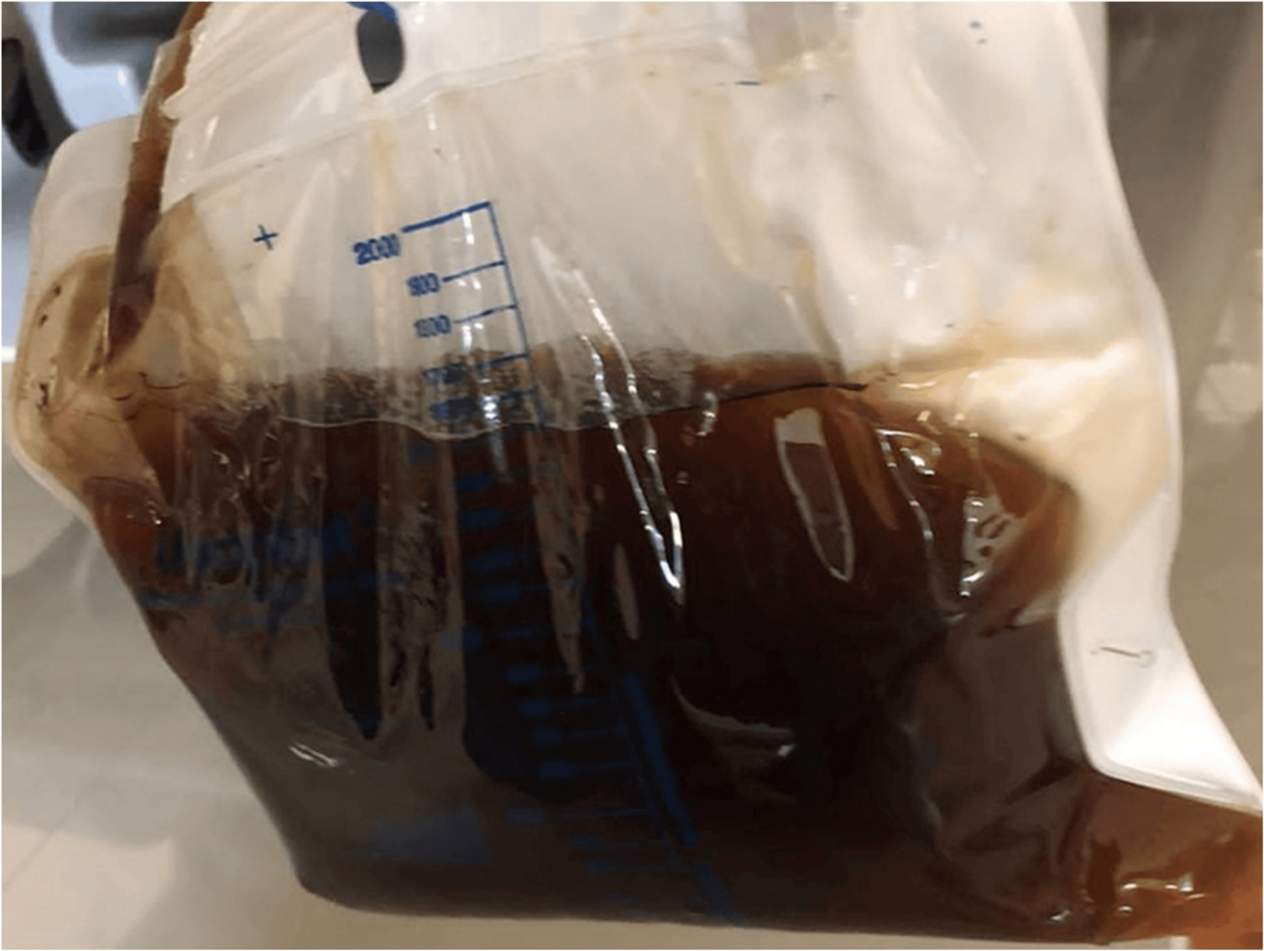
Patient's Urine Collection Bag.

**Figure 3:**
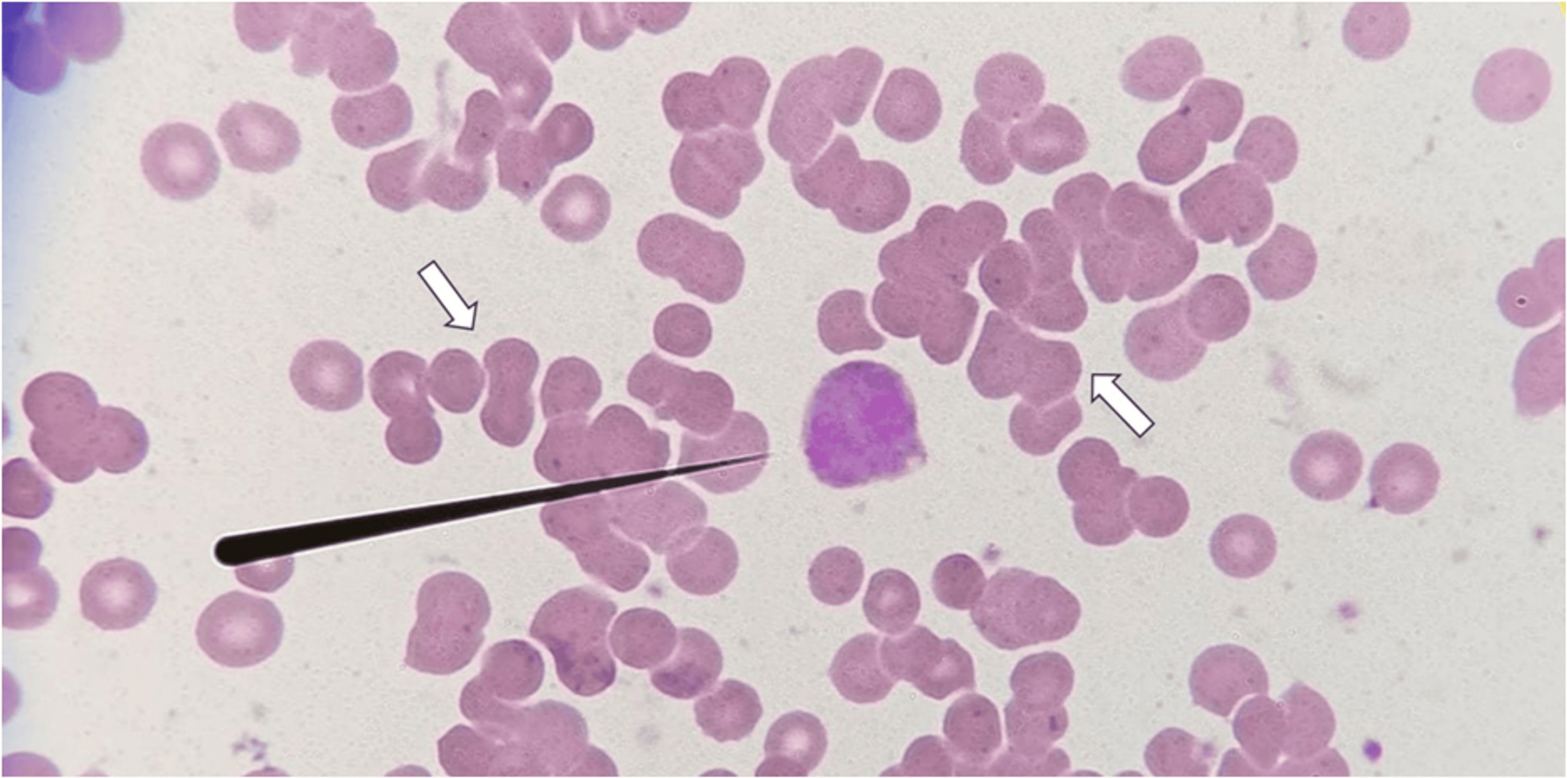
Wright Stain of Peripheral Blood Smear. Narrow black line, eosinophil; white arrow, red blood cell agglutination.

Following the surgical procedure, the patient experienced a sudden decline in neurological and respiratory function, necessitating advanced airway management and the administration of vasopressors. Jaundice and choluria were observed (Figure 2). Cerebral ischemia or hemorrhage and pulmonary thromboembolism were ruled out. Two doses of methylprednisolone were administered due to the suspected reactivation of ANCA-negative vasculitis in the lungs. Despite these interventions, there was a progressive reduction in hemoglobin levels with an increase in lactate dehydrogenase and total bilirubin. Positive DAT IgG/C3d and red blood cell agglutination in peripheral blood smear were detected (Figure 3). Consequently, immunohematology tests were conducted to investigate the cause of hemolysis (Table 1), leading to the diagnosis of PCH.

**Table 1:** Immunohematology Tests.

Test	Result
Direct antiglobulin IgG/C3d	Positive 4+
Direct antiglobulin IgG	Negative
Direct antiglobulin C3d	Positive 4+
Cold agglutinins titer	>1:512
Donath-Landsteiner test	Positive
Red blood cell antibody screen	Negative
Serum protein electrophoresis	Polyclonal pattern
Serum immunofixation	Polyclonal pattern

**Figure 4:**
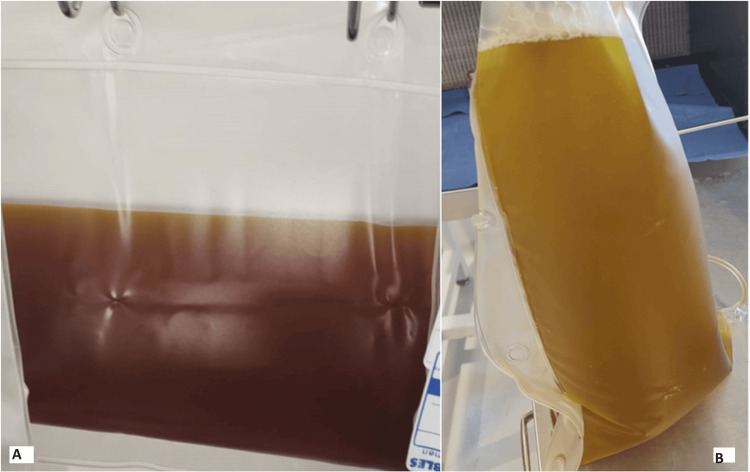
Plasmapheresis Waste Bag. Hemolysis is noted in the waste bag. A: First PEX; B: last PEX. PEX: Plasma exchange

Bridging therapy was initiated, consisting of IVIg at 0.4 mg/kg/day and 2 grams of MMF per day. This was followed by PEX (Figure 4) on the 20th day of hospitalization at intervals of every 48 hours at 1.0 volumes. Additionally, a single dose of 1 gram of Cy was administered on the 22nd day, and intravenous RTX at a dosage of 375 mg/m^2^ was given on the 24th day. Anti-complement therapy was not available. Due to a lack of response to pulmonary protective measures, and empirical antibiotic therapy, a bronchoscopy was performed again before the second PEX, resulting in another episode of hemolysis. On the 25th day of hospitalization, sedation was discontinued, and the patient had a calm awakening. This was followed by successful weaning from vasopressors, maintenance of perfusion pressure, and minimal ventilatory parameters in spontaneous mode through a tracheostomy.

He maintained saturation goals at 96%, tolerated enteral nutrition, and had adequate urine output. Two days later, he developed febrile neutropenia secondary to Cy and an infection with Acinetobacter baumannii, ultimately succumbing 48 hours later.

## Discussion

PCH represents less than 1% of autoimmune hemolytic anemia (AIHA) cases, with a global incidence of 0.04 cases per 100,000 inhabitants without gender predominance [6]. Half of the cases occur in individuals under five years old, with a second peak of incidence after the age of 50 [5,6], although this may vary depending on the studied population. For example, Zeller et al. reported 14 positive DLT tests in children and 3 in adult patients in the Canadian laboratory database over a 30-year period [7]. In Germany, PCH accounted for 4.1% of the 531 studied AIHA cases, all of which were pediatric patients [8]. In the Mediterranean, the GIMEMA group reported only 1 case of PCH in a series of 308 patients with AIHA [9].

So far, no cases have been reported in Mexico [6-8]. Generally, patients present with upper respiratory tract infections, both viral (Influenza A) and bacterial (Staphylococcus aureus, Streptococcus pneumoniae, Mycoplasma pneumoniae, Haemophilus influenzae), gastroenteritis (the patient did not present with diarrhea or symptoms suggestive of gastrointestinal infection), cytomegalovirus, Epstein-Barr virus, Klebsiella pneumoniae, Escherichia coli, measles, rubella, varicella-zoster, adenovirus, and syphilis. All were ruled out through cultures, serological tests, and PCR [6-9].

The reported case involved a patient from Monterrey, Mexico, a city with a warm and semi-arid climate, resulting in limited exposure to cold throughout his life. In January 2020, during the winter, he was diagnosed with ANCA-negative vasculitis with pulmonary and renal involvement, along with pauci-immune glomerulonephritis, which improved with immunosuppression, hemodialysis, and PEX. Concurrently, he experienced refractory AIHA, evidenced only by a positive polyspecific DAT, which also improved with the treatment of the vasculitis. Additionally, family members reported that he selectively avoided artificial low temperatures due to discomfort, which may have contributed to clinical bias, given the rarity of the disease, limited access to specialized immunohematology tests, and the lack of clinical suspicion at the time [10].

## Conclusions

This is the first reported case of PCH in Mexico. The largest series of reported cases originate from countries with latitudes closer to the poles, where physicians encounter this disease more frequently, enabling timely diagnosis and treatment. Unfortunately, our case had a fatal outcome due to a delayed diagnosis, as the test was sent to the United States, and due to complications with the available treatment. This highlights the importance of involving a hematologist specializing in transfusion medicine in the study of AIHA in tropical climates and ensuring that immunohematology tests are readily available at a regional level for timely diagnosis.
